# Effect of therapeutic exercise on the balance of patients with progressive supranuclear palsy: A pilot study

**DOI:** 10.3389/fneur.2022.955893

**Published:** 2022-09-13

**Authors:** Naomi Matsuda, Yasuyuki Takamatsu, Ikuko Aiba

**Affiliations:** ^1^Department of Rehabilitation, National Hospital Organization Higashinagoya National Hospital, Nagoya, Japan; ^2^Department of Rehabilitation Science, Faculty of Health Sciences, Hokkaido University, Sapporo, Japan; ^3^Department of Neurology, National Hospital Organization Higashinagoya National Hospital, Nagoya, Japan

**Keywords:** progressive supranuclear palsy, exercise, rehabilitation, rehabilitation research, balance

## Abstract

**Background:**

Progressive supranuclear palsy (PSP) is a parkinsonian-like progressive neurodegenerative syndrome. Key clinical features include ocular motor dysfunction, postural instability, and cognitive dysfunction. Maintaining and improving balance function and gait function are very important for patients with PSP with severe postural dysfunction and repeated falls. In addition, patients with PSP have a poor response to pharmacological treatment; hence, rehabilitation is a key approach in dealing with this syndrome. However, no conclusion on the beneficial effects of rehabilitation for patients with PSP have been established in the literature.

**Objectives:**

The effectiveness of multiple therapeutic exercise program with probable or possible PSP according to the Movement Disorder Society criteria for the clinical diagnosis of PSP was validated.

**Methods:**

Participants underwent multiple therapeutic exercise program customized for each participant, including resistance training, balance training, and walking exercises that were performed for 60–80 minutes a day, 5 days a week for 4 weeks. The outcomes measured were as follows: pull test, Berg Balance Scale (BBS), timed up and go test (TUG), and gait speed test.

**Results:**

A total of 117 patients with PSP were enrolled and the analysis was performed on 20 patients with probable PSP. Four-week rehabilitation significantly improved pull test (*p* = 0.034) and BBS scores (p = 0.001). There were no significant differences both TUG (*p* = 0.502) and gait speed (*p* = 0.813).

**Conclusion:**

The multiple therapeutic exercise program had beneficial effects on balance performance in patients with PSP in 4 weeks and could be an essential element in their rehabilitation. Although this pilot study was conducted without a control group, it provided valuable information for future prospective randomized controlled trials.

## Introduction

Progressive supranuclear palsy (PSP) is a parkinsonian-like progressive neurodegenerative syndrome. Key clinical features include ocular motor dysfunction, postural instability, akinesia, and cognitive dysfunction (1, 2). In addition, mobility problems are the most common early feature in PSP, and onset of falls within 1 year has been associated with a worse prognosis (3).

Maintaining and improving balance function and gait function are very important for patients with PSP with severe postural dysfunction and repeated falls. Rehabilitation for idiopathic Parkinson's disease (PD) was proven to be effective for improving the motor and balance function (4–6). Furthermore, rehabilitation combined with pharmacotherapy is known to improve motor symptoms, gait, and quality of life of patients with PD (7, 8). However, patients with PSP respond poorly to pharmacological treatment (2); consequently, rehabilitation could be highly relevant for maintaining the motor function and activities of daily living (ADL) in PSP (9).

Several studies related to rehabilitation interventions for patients with PSP used therapies designed for them. These therapies included balance and eye movement training (10, 11), harness-supported treadmill training (12–14), weighted vests during ambulation (15), and robot-assisted gait training (12, 16), of these, balance exercise (10, 11) and gait training (12–14, 16) indicated potential benefit. However, a recent systematic review concluded that the effects of structured physical activity for patients with advanced PSP remain unknown (17).

Moreover, the impact on patients with PSP of different multiple therapeutic exercise program (including resistance training, balance training, walking exercises) effective for motor and balance function in the elderly and patients with PD has not been investigated in a sufficient manner yet.

Our study aimed to determine whether multiple patient-specific customized therapeutic exercise program are effective in improving balance and motor functions of patients with PSP.

## Methods

### Participants

We conducted a pre–post study between January 2016 and December 2021 that included probable or possible patients with PSP according to the Movement Disorder Society criteria for the clinical diagnosis of PSP (2) in the National Hospital Organization Higashinagoya National Hospital. The exclusion criteria were: (1) a score of the modified Rankin Scale (mRS) ≥ 5, (2) patients with subtypes except Richardson syndrome (RS), (3) patients who underwent rehabilitation treatment for 4 consecutive weeks in the past, (4) inability to walk without assistance for at least 10 meters, (5) patients who were discharged within 4 weeks, and (6) patients with missing values in the outcome data. Patients were hospitalized for the purpose of rehabilitation. The medication of all participants was not changed to maintain a stable condition during the rehabilitation period.

### Measured outcomes

The age, disease duration, sex, subtype, frontal assessment battery (FAB), mini-mental state examination (MMSE), mRS, and the PSP rating scale (PSPRS) scores of all patients were evaluated (18). The balance and basic motor functions were evaluated using the Berg Balance Scale (BBS) (19), timed up and go test (TUG) (20), and gait speed. All patients were evaluated for balance and basic motor functions within 2 days before and 4 weeks after rehabilitation. Participants underwent the following tests:

(1) PSPRS (18): The PSPRS was developed to assess disease severity in patients with PSP. Furthermore, PSPRS assesses characteristic symptoms associated with PSP, including behavioral change, ocular-motor, gait, and balance disfunctions. The maximum total score is 100 points. Higher scores indicate high disease severity. The PSPRS subitem scores and total score were evaluated as baseline, and the scores of V: limb movements and VI: gait and midline were evaluated pre and before interventions.
(2) Pull test (18, 21, 22): The pull test is used for evaluating postural stability (0–4 points) as a component of PSPRS. The examiner stands behind the patient and applies a strong pull on the shoulders with the patient erect with eyes open and feet comfortably apart.
(3) BBS (19): This evaluated the individual's balance abilities during the performance of 14 items (0–4 points per item), such as sitting, standing, and one leg standing, and positional changes. The maximum total score is 56 points. Higher scores indicate good balance ability.
(4) TUG (20): This evaluation consisted of the participant standing up from a sitting position in the chair with a seat height of 40 cm, walking a distance of 3 m, then passing around a cone, returning, and sitting back down in the chair. Comfortable and maximum gait speeds were both measured once each. To assess comfortable speed walking, participants were instructed to walk at their normal comfortable (natural) speed. To assess maximum walking speed, they were asked to walk as fast as possible without running.
(5) Gait speed: Comfortable and maximum gait speed were both measured once each. Participants were required to accelerate and decelerate 2 m before and after the 10 m test distance. To assess comfortable speed walking, participants were instructed to walk at their normal comfortable speed. To assess maximum walking speed, they were asked to walk as fast as possible without running.

This study was not considered about an assessment bias, therefore it included cases that the evaluators are the same professionals in charge of performing the therapies.

### Intervention programs

The multiple therapeutic exercise program consisted of balance training, resistance training, range of motion (ROM) exercises, stretching, walking exercises, and ADL training and was customized for each patient by physical and occupational therapists. More details about multiple therapeutic exercise program are provided in Supplementary Table 1. The program was performed for 60–80 mins a day, 5 days a week for 4 weeks.

### Statistical analysis

We evaluated the normality of the distribution of all variables using the Shapiro–Wilk test. For comparison before and after the rehabilitation, we used the Wilcoxon signed-rank test or paired *t*-tests. In addition, we calculated the effect size (r) by the test statistic. Data were reported as mean ± standard deviation for normally distributed data and number for discrete variables. We performed the statistical analysis using the SPSS software, version 26 (IBM Inc., Armonk, NY, USA). A *p*-value of <0.05 was considered statistically significant.

## Results

**Figure 1:**
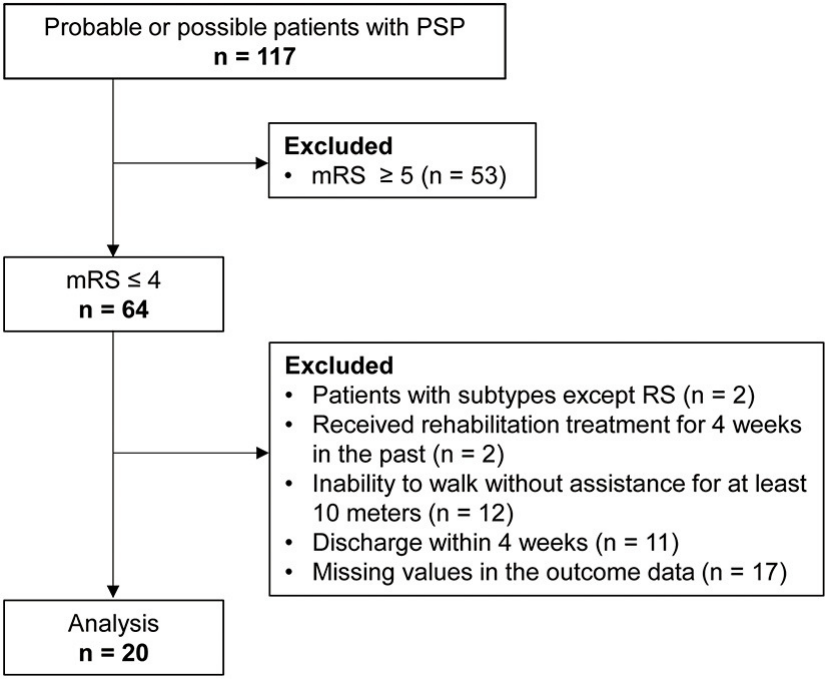
Flow chart.

We enrolled 117 probable or possible patients with PSP. We excluded the following patients who met the exclusion criteria: (1) a score of mRS ≥ 5, *n* = 53; (2) patients with subtypes except RS, *n* = 2; (3) patients who underwent rehabilitation treatment for 4 weeks in the past, *n* = 2; (4) inability to walk without assistance for at least 10 m, *n* = 12; (5) patients who were discharged within 4 weeks, *n* = 11; and (6) patients with missing values in the outcome data, *n* = 17. Consequently, the analysis was performed on 20 patients with probable PSP (Figure 1).

Table 1 lists the participants' demographic and clinical characteristics.

**Table 1:** Demographic and clinical characteristics of participants.

	***N* = 20**
Age, years	72.3 ± 6.2 (61–83)
Disease duration, months	29.3 ± 18.2 (10–82)
Sex, men/women	13/7
FAB, score	12.2 ± 3.2 (6–17)
MMSE, score	26.0 ± 2.6 (22–30)
mRS, score	3.3 ± 0.6 (2–4)
**PSPRS, score**	
I	7.3 ± 2.4 (3–11)
II	3.2 ± 2.4 (0–8)
III	2.7 ± 1.2 (1–5)
IV	6.7 ± 3.9 (0–12)
V	3.9 ± 1.3 (2–7)
VI	8.2 ± 2.1 (6–12)
Total	31.8 ± 10.0 (16–50)

Data were reported as mean ± standard deviation (minimum—maximum) and numbers.

Table 2 shows the results of PSPRS V: Limb motor and VI: Gait and midline pre (0W) and post (4W) rehabilitation. Four-week rehabilitation significantly improved V: Gait and midline total scores (*p* = 0.004, *r* = 0.645). Similarly, significant improvements were observed in the VI subitems of arising from chair (*p* = 0.007, *r* = 0.607), and postural stability (*p* = 0.034, *r* = 0.474).

**Table 2:** Each subitem (V: Limb motor and VI: Gait and midline) score of PSPRS pre (0W) and post (4W) rehabilitation.

		**Pre**	**Post**	***p*-value**	**Effect size**
V. Limb motor	Limb rigidity (full score = 4)	1.4 ± 0.9 (0–3)	1.3 ± 0.9 (0–3)	0.655	0.100
	Limb dystonia (full score = 4)	0.3 ± 0.6 (0–2)	0.3 ± 0.5 (0–1)	1.000	0.000
	Finger tapping (full score = 2)	0.9 ± 0.5 (0–2)	0.8 ± 0.5 (0–2)	0.564	0.129
	Toe tapping (full score = 2)	1.0 ± 0.4 (0–2)	0.9 ± 0.4 (0–2)	0.317	0.224
	Apraxia of hand movement (full score = 2)	0.1 ± 0.3 (0–1)	0.1 ± 0.2 (0–1)	0.317	0.224
	Tremor in any part (full score = 2)	0.3 ± 0.4 (0–1)	0.3 ± 0.5 (0–2)	1.000	0.000
	Total (full score = 16)	3.9 ± 1.6 (2–7)	3.6 ± 1.9 (0–7)	0.406	0.186
VI.Gait and midline	Neck rigidity or dystonia (full score = 16)	1.4 ± 0.9 (0–3)	1.3± 0.9 (0–3)	0.083	0.387
	Arising from chair (full score = 4)	1.7 ± 0.7 (1–3)	1.2 ± 0.8 (1–3)	0.007*	0.607
	Gait (full score = 4)	1.7 ± 0.7 (1–3)	1.5 ± 0.7 (1–3)	0.102	0.365
	Postural stability (full score = 4)	2.0 ± 0.8 (1–3)	1.7 ± 0.8 (1–3)	0.034*	0.474
	Sitting down (full score = 4)	1.5 ± 0.5 (1–2)	1.3 ± 0.6 (1–2)	0.102	0.365
	Total (full score = 20)	8.2 ± 2.1 (5–12)	6.9 ± 2.6 (5–11)	0.004*	0.645

Data were reported as mean ± standard deviation (minimum—maximum). *Indicates p < 0.05.

Table 3 shows the results of the motor function pre (0W) and post (4W) rehabilitation, and the plot data are shown in Supplementary Figure 1 in the Supplemental material. Four-week rehabilitation significantly improved pull test results (*p* = 0.034, *r* = 0.474, Supplementary Figure 1A) and BBS total scores (*p* = 0.001, *r* = 0.679, Supplementary Figure 1B). There were no significant differences between pre and post in TUG (comfortable: *p* = 0.502, *r* = 0.150; maximum: *p* = 0.956, *r* = 0.013, Supplementary Figure 1C) and gait speed (comfortable: *p* = 0.813, *r* = 0.055; maximum: *p* = 0.566, *r* = 0.133, Supplementary Figure 1D). These results suggest that 4-week rehabilitation improved postural control and balance function in PSP.

**Table 3:** Motor function pre (0W) and post (4W) rehabilitation.

		**Pre**	**Post**	***p*-value**	**Effect size**
Pull test, score		2.0 ± 0.8 (1–3)	1.7 ± 0.8 (0–3)	0.034*	0.474
BBS, score		40.6 ± 8.2 (20–52)	44.6± 8.5 (20–56)	0.001*	0.679
TUG, sec	Comfortable	15.3 ± 3.7 (10.6–26.4)	14.9 ± 3.5 (10.4–22.0)	0.502	0.150
	Maximum	12.5 ± 3.1 (8.0–20.2)	12.5 ± 2.9 (8.4–20.1)	0.956	0.013
Gait speed, m/min	Comfortable	56.6 ± 12.9 (28.3–83.3)	57.4 ± 13.4 (26.4–82.8)	0.813	0.055
	Maximum	74.8 ± 13.8 (48.9–113.0)	73.3 ± 16.6 (43.8–111.7)	0.566	0.133

Data were reported as mean ± standard deviation (minimum—maximum).

^*^Indicates p < 0.05.

Table 4 shows the results of each subitem of BBS pre (0W) and post (4W) rehabilitation, and the plot data are shown in Supplementary Figure 2 in the Supplemental material. There were significant improvements in the items of reaching forward with outstretched arm (*p* = 0.011, *r* = 0.566), turning to look behind (*p* = 0.039, *r* = 0.461), turning 360 degrees (*p* = 0.046, *r* = 0.447), standing with one foot in front (*p* = 0.047, *r* = 0.445), and standing on one foot (*p* = 0.009, *r* = 0.588).

**Table 4:** Each subitem score of Berg Balance Scale pre (0W) and post (4W) rehabilitation.

	**Pre**	**Post**	***p*-value**	**Effect size**
Sitting to standing	3.4 ± 0.8 (2–4)	3.6 ± 0.8 (1–4)	0.453	0.168
Standing unsupported	3.5 ± 0.5 (3–4)	3.7 ± 0.5 (3–4)	0.102	0.365
Sitting unsupported	4.0 ± 0.0 (4–4)	4.0 ± 0.0 (4–4)	1.000	0.224
Standing to sitting	3.5 ± 0.7 (2–4)	3.5 ± 1.0 (0–4)	0.705	0.085
Transfers	3.0 ± 0.8 (1–4)	3.2 ± 0.8 (1–4)	0.157	0.316
Standing with eyes closed	3.6 ± 0.5 (3–4)	3.5 ± 0.5 (3–4)	0.564	0.129
Standing with feet together	3.3 ± 0.7 (1–4)	3.5 ± 0.6 (2–4)	0.257	0.254
Reaching forward with outstretched arm	3.1 ± 1.1 (0–4)	3.6 ± 0.9 (0–4)	0.011*	0.566
Retrieving object from floor	3.0 ± 1.1 (0–4)	3.1 ± 1.1 (0–4)	0.083	0.387
Turning to look behind	3.4 ± 1.1 (0–4)	3.9 ± 0.5 (2–4)	0.039*	0.461
Turning 360 degrees	1.8 ± 1.0 (0–4)	2.3 ± 1.0 (0–4)	0.046*	0.447
Placing alternate foot on stool	2.0 ± 1.4 (0–4)	2.3 ± 1.3 (0–4)	0.165	0.310
Standing with one foot in front	2.0 ± 1.4 (0–4)	2.5 ± 1.2 (0–4)	0.047*	0.445
Standing on one foot	1.2 ± 1.0 (0–3)	2.0 ± 1.4 (0–4)	0.009*	0.588

Data were reported as mean ± standard deviation (minimum—maximum).

^*^Indicates p < 0.05.

## Discussion

The present study shows that multiple therapeutic exercise program can improve the balance function in patients with PSP. As no standard pharmacological treatment them has been established yet (recall that PSP has poor response to Levodopa) (9, 23), therapeutic exercise is a key non-pharmacological approach for maintaining their motor function. However, the eventual positive effects of therapeutic exercise in patients with PSP have been insufficiently documented yet (17); therefore, our investigation is among the first to reveal the effectiveness of therapeutic exercise for these patients. This study excluded a control group. Therefore, this limitation hinders the applicability of the results. However, this pilot study provided valuable information for future prospective randomized controlled trials.

### PSPRS

In PSP with similar severity, rehabilitation interventions improve PSPRS items V: Limb motor and VI: Gait and midline (12, 13). In this study, multiple therapeutic exercises improved items VI and VI subitems; arising from chair and postural stability post-intervention. Therefore, rehabilitation was expected to enhance the stability of gait and basic movements such as standing, sitting, etc., in patients with PSP.

### Balance functions

The pull tests and BBS provided useful measures for changes that are related to balance function. The pull test easily evaluates postural instability and predicts falls in PD (21, 24). The BBS is a wellaccepted, comprehensive evaluation of balance that has excellent reliability and validity with older adults (25). It can also continuously monitor balance function and predict falls in patients with PD and neurodegenerative diseases (26, 27).

In the pre-intervention evaluation of balance function, the pull test showed that the participants had moderate postural instability. The total BBS score of participants who had moderate balance dysfunction was 40.6. In PD, the fall risk cutoff score on BBS reported by Dibble was 54 of 56 (28), that reported by Landers was 44 (29); the participants of this study had a higher fall risk. The results of the BBS subitems (Table 4) showed particularly low values for turning 360 degrees, placing alternate foot on a stool, standing with one foot in front, and standing on one foot. These results suggest that participants have particularly impaired anticipatory postural adjustments (APA), reactive postural adjustments (RPA) and the more challenging balance items. The main lesions of PSP are the substantia nigra, subthalamic nucleus, brain stem, globus pallidus, tegmental portion pons, subthalamic nucleus, cerebellar dentate nucleus, and frontal lobe (1, 30). Postural instability is the primary symptom of PSP, which impairs the ability to maintain balance while standing, rising, seated, changing direction, and walking, making the patient susceptible to falls. The balance dysfunction observed in patients with PSP is primarily a disruption of reactive and anticipatory postural coordination and is associated with dysfunction related to the basal ganglia and brainstem. In addition, cerebellar disturbances may potentiate postural instability.

A few recent reports have assessed the effect of several rehabilitation programs (12–14, 31, 32) for balance function in PSP. In the post-intervention assessment of balance function, our multiple therapeutic exercise program were found to improve balance function in patients with PSP, similar to earlier studies.

Several subsystems contribute to postural stability, such as the functional level of motor systems, anticipatory postural control, dynamic stability, static stability, sensory integration, functional stability limits, reactive postural control, cognitive influences, and verticality (33). Balance training was conducted, including exercise to cope with postural changes when moving the body voluntarily as an intervention for APA disorders and exercise to control posture against external disturbances as an intervention for RPA disorders. In addition, ROM exercises adjusted the participants' optimal postural alignment by improving ROM of limited joints, and strength training focusing on antigravity muscles was performed to maintain posture. These multiple approaches may have improved the balance function such as RPA, APA, static stability, functional stability limits in patients with PSP.

### Motor functions

The TUG and gait speed provided useful measures for changes that are related to motor function and the risk of fall. A previous study reported fall risk cutoff score for TUG comfortable time to be 11.5 s in PD (34), whereas another reported it to be 13.5 s in elderly persons (35); the score in this study was 15.3 s. The fall risk cutoff score reported for comfortable 10-m gait speed was 1.1–1.2 m/sec in PD (36) and 1.0 m/sec in elderly persons (37), whereas the score in the present study was 56.6 m/min (converted value 0.94 m/sec). In the pre-intervention evaluation of motor function, participants' motor function was found to be lower than both patients with PD and elderly persons, who were at a higher risk for falls.

In patients with PD, therapeutic exercise, including ROM, stretching, balance training, resistance training, and treadmill walking, was effective for improving motor function, muscle strength, balance function, and gait speed (4, 8). The effect of the same multiple therapeutic exercise program was found to be beneficial for balance function in patients with PSP as well; however, their gait function did not improve as expected.

The fall risk cutoff score for both TUG and gait speed suggests that greater speed is associated with a lower risk of falls in patients with PD and the elderly. In contrast, as postural instability appears in the early stages in patients with PSP, it leads to difficulties in balance control and thereby to an increased risk of falls. As the disease progresses, gait instability due to cerebellar damage is observed in addition to symptoms of parkinsonism, such as rush symptoms and frozen gait (38). Therefore, we argue that primary rehabilitation for patients with PSP should focus on improving their gait stability instead of their gait speed. This study did not assess gait stability; therefore, evidence on the effects of gait stability on fall reduction needs to be validated in the future.

### Limitations

This study has several limitations. First is the fact that it was a retrospective study in a single facility involving a small sample size, the lack of a control group, and absence of follow-ups. Further prospective multicenter studies with a larger sample size, randomized controlled trial setting, and follow-up of long-term rehabilitation can help validate and support our findings. Second, in some cases, the intervention and evaluation were conducted by the same therapists, and there might be concerns about the assessment bias. Therefore, pre-determination and arrangement are needed in which intervention and evaluation are not performed by the same therapists in further prospective studies. Lastly, we lacked the assessment of the quality of life (QoL). This study mainly focused on motor and balance functions of PSP, but the impact of the therapeutical intervention on patients' QoL should be known. In the future, the effect of rehabilitation on QoL of patients with PSP by PSP-QoL (39) or EQ-5D (40) should be investigated.

## Conclusion

In this pre–post study, multiple 4-week long therapeutic exercise program known to improve several functions of patients suffering from PD were shown to induce beneficial effects on balance function in patients with PSP.

## Data availability statement

The original contributions presented in the study are included in the article/Supplementary material, further inquiries can be directed to the corresponding author/s.

## Ethics statement

The studies involving human participants were reviewed and approved by the Ethics Committee of the National Hospital Organization Higashinagoya Hospital (approval number 30-11). Written informed consent from the patients/participants or patients/participants' legal guardian/next of kin was not required to participate in this study in accordance with the national legislation and the institutional requirements. All participants were verbally informed of the study and their consent was obtained.

## Author contributions

NM and YT prepared and repeatedly revised the manuscript. IA is responsible for the ethics application and contributed to the development, procedure, and funding for research. YT and IA revised the manuscript. All authors reviewed the manuscript and provided final approval for the manuscript.

## Funding

This work was supported by Grants-in Aid from the Research Committee of CNS Degenerative Diseases, Research on Policy Planning and Evaluation for Rare and Intractable Diseases, Health, Labor, and Welfare Sciences Research Grants, the Ministry of Health, and Labor and Welfare, Japan (20FC1049 to IA).

## Conflict of interest

The authors declare that the research was conducted in the absence of any commercial or financial relationships that could be construed as a potential conflict of interest.

## Publisher's note

All claims expressed in this article are solely those of the authors and do not necessarily represent those of their affiliated organizations, or those of the publisher, the editors and the reviewers. Any product that may be evaluated in this article, or claim that may be made by its manufacturer, is not guaranteed or endorsed by the publisher.
