# Supplementary material for: Effect of therapeutic exercise on the balance of patients with progressive supranuclear palsy: A pilot study
Source: Front Neurol. 2022 Sep 13;13:955893. doi: 10.3389/fneur.2022.955893 (PMC9513196; doi:10.3389/fneur.2022.955893)
Supplement: Supplementary file 1 [file Data_Sheet_1.PDF]

Supplementary Material

1 Supplementary Table

Supplementary Table 1. Multiple therapeutic exercise program

Program	Detailed description of programs
Balance training	• Moving the center of gravity back, forth, left, or right in sitting position<br>• Quadruped leg lift in all-fours position<br>• Holding upper limb elevation or rotating the trunk in kneeling and half-kneeling positions<br>• Holding standing position on a stable or an unstable surface in opened leg standing, closed leg standing, tandem standing or one leg standing<br>• Reaching back, forth, left, or right<br>• Stepping back, forth, left, or right<br>• Turning clockwise or anticlockwise<br>• Balance walking: tandem walking, side step, cross step, backward walking
Muscle strength training	Loading by the patient's own weight or by the hand of the therapist on limbs, neck, and trunk.<br>• Leg Raise<br>• Bridges<br>• Crunch<br>• Squats<br>• Heel and toe raises<br>• Front lunges<br>• Side lunges
Range of motion exercise and stretching	Limbs, neck, and trunk assistive or passive range of motion exercise by the therapist
Gait training	• Sudden starts and stops<br>• Walking and turning around<br>• Shuttle walking<br>• Side walking<br>• Tandem walking<br>• Cross-step<br>• Backward walking<br>• Treadmill training
Activities of daily living	• Transfer activities: getting up from the bed, rising up from the chair, and sitting down in the chair, standing, walking indoors and

	outdoors<br>・Toilet activities: getting to and from the toilet, turning in the toilet, and cleaning oneself.<br>・Bathing activities: transferring in the bathroom, straddling the bathtub, washing one’s face, hair, and body.

2 Supplementary Figures

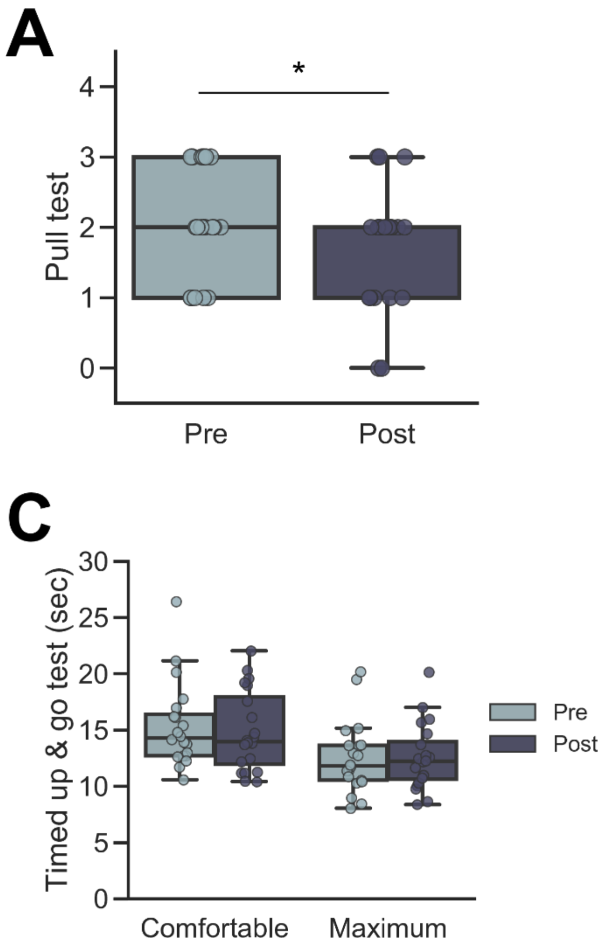

Supplementary Figure 1. The results of the pull test (A), Berg Balance Scale (B), timed up and go test (C) and gait speed (D) at pre and post. * indicates $p < 0.05$.

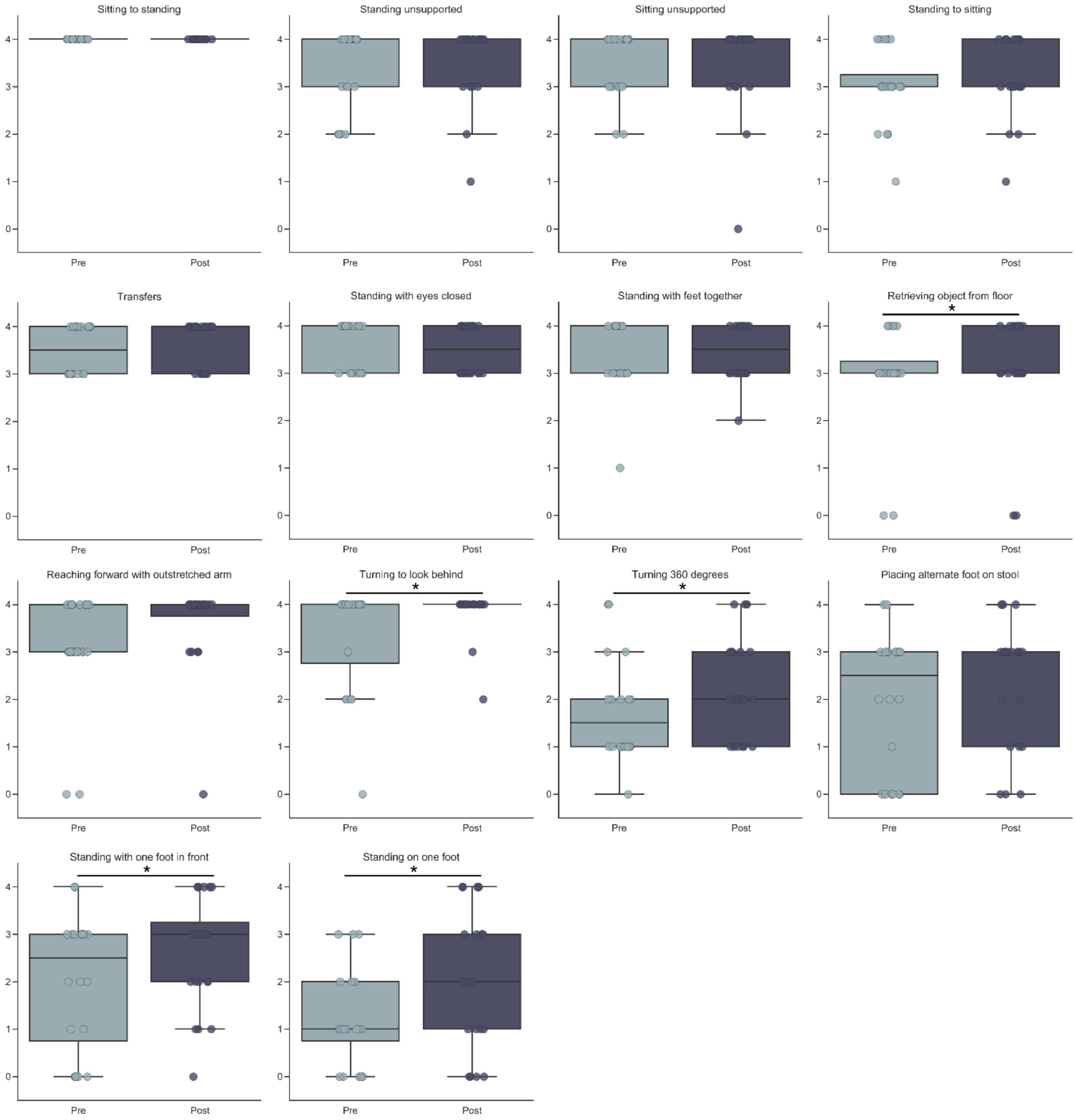

Supplementary Figure 2. The results of each subitem of Berg Balance Scale at pre and post. * indicates $p < 0.05$.
